# Parental Social Environment Has no Effect on Offspring Development in the Dung Beetle: A Test of Adult Sex Ratio Effects

**DOI:** 10.1002/ece3.73833

**Published:** 2026-07-06

**Authors:** Lisheng Zhang, Sudeshna Chakraborty, Tamás Székely, Jan Komdeur

**Affiliations:** ^1^ Yunnan Key Laboratory of Forest Ecosystem Stability and Global Change, Xishuangbanna Tropical Botanical Garden Chinese Academy of Sciences Mengla Yunnan China; ^2^ Behavioural and Physiological Ecology Group Groningen Institute for Evolutionary Life Science, University of Groningen Groningen the Netherlands; ^3^ Milner Centre for Evolution University of Bath Bath UK; ^4^ HUN‐REN‐DE Reproductive Strategies Research Group University of Debrecen Debrecen Hungary

**Keywords:** adult sex ratio, dung beetle, offspring development, parental care, social environment, transgenerational plasticity

## Abstract

The adult sex ratio (ASR) is a key demographic parameter that shapes sexual selection and social interactions. While ASR variation drives profound behavioral plasticity within a generation, it remains unclear whether parental experience of skewed ASRs influences offspring development via transgenerational plasticity (TGP), and if so, which of the two core nongenetic pathways: pre‐zygotic germline‐mediated information transfer and post‐zygotic parental investment, predominates. To disentangle these mechanistically distinct pathways, we conducted a controlled egg‐transplantation experiment in the dung beetle *Onthophagus taurus*. We exposed parental beetles to female‐biased, unbiased, and male‐biased social environments, first confirming that our ASR manipulations generated the predicted gradients of social stress by quantifying contest and courtship behaviors. We then transplanted eggs from naturally produced brood balls across different ASR treatments into standardized artificial brood balls to strictly isolate germline‐mediated TGP effects, while weighing original dried brood ball mass to independently assess parental investment. Our results revealed a striking dissociation between parental behavioral responses and intergenerational outcomes. ASR strongly modulated adult social interactions: male‐biased treatments exhibited the highest contest intensity, whereas female‐biased treatments displayed significantly higher courtship frequency than both unbiased and male‐biased groups. Despite these pronounced parental adjustments, parental ASR experience had no significant effect on any measured offspring developmental trait, including developmental speed, emergence weight, and stage‐specific developmental duration. Concurrently, parental investment (dried brood ball mass) did not differ across ASR treatments. These findings demonstrate that ASR‐induced social pressures do not propagate to offspring metamorphic development via either core TGP pathway within a single generation, suggesting that offspring development is strongly canalized against parental social fluctuations. Future research should disentangle cryptic non‐nutritional transmission pathways (e.g., microbiome inoculation) and employ gametic epigenetic assays to determine if social experiences leave molecular traces under alternative ecological contexts or longer evolutionary timescales.

## Graphical abstract

We investigated whether adult sex ratio (ASR) induces transgenerational plasticity in the dung beetle *Onthophagus taurus* via germline transmission or behavioral parental investment. Although varying ASR treatments significantly altered parental contest and courtship behaviors, utilizing a novel egg‐transplantation protocol revealed no ASR‐induced effects on offspring developmental trajectories or brood ball mass. These findings indicate that while adult dung beetles exhibit profound behavioral plasticity to local social demography, offspring development remains strictly canalized against such social fluctuations over short timescales.

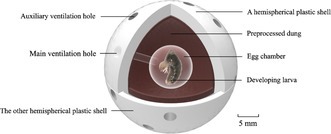

## 1 Introduction

The adult sex ratio (ASR), defined as the proportion of adult males to females in a population, is a fundamental demographic parameter varying widely across species and ecological contexts (Székely et al. 2014). Most ASR research has focused on its intragenerational consequences, demonstrating that it shapes mating systems, sexual selection, and reproductive strategies (Schacht et al. 2017, 2022). However, parental social environment responses also influence offspring phenotypes via transgenerational plasticity (TGP), defined as environment‐induced nongenetic parental effects independent of transmission pathway (McAndry et al. 2025). Male‐biased ASR intensifies competition and sexual harassment, triggering widespread endocrine and physiological costs. Such high conflict drastically reduces female survival and fecundity via elevated stress hormones (Aspbury et al. 2017) and toxic seminal fluids (Wigby and Chapman 2004), while driving rapid metabolic exhaustion and male mortality from extreme, hormonally‐mediated reproductive effort (Ros et al. 2003; McKermitt et al. 2024). Conversely, female‐biased ASR reduces male mating opportunities, selecting for increased paternal investment to ensure paternity (Kokko and Jennions 2008). Because ASR simultaneously modulates such mating‐parenting trade‐offs and parental physiological states, it should theoretically trigger TGP through two distinct, non‐mutually exclusive mechanisms: altered post‐zygotic parental investment or pre‐zygotic germline‐mediated nongenetic information transfer. Despite this expectation, it remains unclear which mechanism primarily drives ASR‐induced TGP. Disentangling these pathways is critical for understanding the cross‐generational evolutionary consequences of skewed sex ratios.

Offspring phenotypes are shaped by two fundamentally distinct pathways of parental influence: genetic inheritance, involving the transmission of nuclear DNA sequence variants (Alberts et al. 2022), and transgenerational plasticity (TGP), defined as any phenotypic change in offspring triggered by parental environmental exposure, independent of changes to the nuclear DNA sequence (Bonduriansky 2021; McAndry et al. 2025). While classical frameworks emphasize genetic transmission, TGP is increasingly recognized as the unifying construct for environment‐induced parental effects (McAndry et al. 2025). TGP encompasses two core, non‐mutually exclusive mechanistic pathways: pre‐zygotic germline‐mediated transmission and post‐zygotic transmission via environmentally induced parental behaviors (Bonduriansky 2021; McAndry et al. 2025). Although widely documented in response to abiotic factors (Joschinski and Bonte 2020; Walsh et al. 2024), social stressors, such as population density and competition, are also potent TGP drivers (Hellmann and Sih 2025), inducing developmental programming through both pathways. For instance, parental crowding in desert locusts (*Schistocerca gregaria*) influences offspring traits via egg chemical deposition (Miller et al. 2008; Simpson et al. 2011). Similarly, maternal exposure to intense male competition in field crickets (*Gryllus bimaculatus*) enhances sons' courtship aggressiveness (Noguera 2023). Crucially, in our focal species, the dung beetle *Onthophagus taurus* , females perceiving high pre‐mating density produce major sons with longer horns independent of dung provisioning, indicating pre‐zygotic transmission (Buzatto et al. 2012). These findings collectively demonstrate that parents perceive social changes and transmit this information via the pre‐zygotic TGP pathway. Alongside germline transmission, TGP operates post‐zygotically via direct parental behaviors like habitat choice and provisioning (Clutton‐Brock 1991; Galef and Laland 2005). This behavioral pathway enhances offspring fitness in holometabolous insects through pupation site selection (Sprague and Woods 2015; Wang et al. 2017) and in matrilineal species through maternally conferred social status (Vullioud et al. 2024).

Parental ASR influences offspring via diverse mechanisms. ASR‐driven sexual selection promotes the transmission of superior genes, consistent with “good genes” theory (Andersson 1994; Qvarnström and Price 2001; Wedell et al. 2002). However, such stable genetic divergence typically accumulates over dozens of generations, as demonstrated in *Drosophila melanogaster* (> 78 generations; Bath et al. 2021) and *Tribolium castaneum* (> 58 generations; Godwin et al. 2020). Evidence for ASR‐induced TGP via germline and behavioral mechanisms is also growing. For instance, male‐biased parental ASR in the non‐caregiving beetle *Callosobruchus maculatus* reduced F0 maternal fitness but transgenerationally enhanced F1 and F2 mating competitiveness, indicating nongenetic environmental priming (Amiri and Bandani 2021). Behaviourally, ASR shapes offspring fitness by altering parental care trade‐offs (Székely et al. 2000). Male‐biased ASRs can increase paternal investment due to limited mating prospects (Kokko and Jennions 2008; Liker et al. 2013), or reduce it due to higher paternity uncertainty (Alonzo 2010; Fromhage and Jennions 2016). Similarly, female‐biased ASRs are expected to increase maternal care (Székely et al. 2000). Unlike classic genetic evolution, TGP transmits environmental cues within a single reproductive cycle, facilitating rapid adaptation. Because our study manipulates ASR across only one generation, sequence‐based evolution cannot explain observed offspring phenotypic differences. We therefore hypothesize that over short timescales, ASR affects offspring development primarily via the dual pathways of TGP, rather than sequence‐based genetic change.

The dung beetle *Onthophagus taurus* (Coleoptera: Scarabaeidae) depends obligately on ephemeral, patchy dung resources for both adult feeding and larval development. Field studies report extreme local densities within individual dung pats: intraspecific densities reach 100–800 individuals/kg dung, with interspecific competitor densities exceeding 1000 individuals/kg (Dadour et al. 1999; Moczek 2003). Notably, natural local ASRs vary dramatically across populations, from strongly female‐biased (♂/♀ ≈ 0.6) in low‐density to strongly male‐biased (♂/♀ ≈ 1.4) in high‐density (Moczek 2003). Although the sensory mechanisms underlying density/ASR assessment and inter‐patch dispersal remain poorly understood, the pervasive effects of ASR across taxa provide a strong a priori rationale for investigating ASR‐mediated TGP in dung beetles. As a holometabolous insect, *O. taurus* undergoes complete metamorphosis through egg, larval, pupal, and adult stages (Gullan and Cranston 2014). Females typically lay one egg per brood ball, provisioned primarily by females with male assistance. Larval development depends entirely on the finite dung resource within the brood ball (Hanski and Cambefort 2014; Scholtz et al. 2009), with nutrition determining male adult size and horn length: well‐fed males develop larger bodies and longer horns, while nutritionally restricted males become smaller with reduced horns (Moczek 2010). Importantly, previous work confirms ASR drives both parental behavior and offspring development in *O. taurus* . Male‐biased populations exhibit elevated contest and courtship intensity (Zhang et al. 2024), indicating skewed ASRs induce social stress that could trigger TGP. Additionally, male presence reduces female brood production, and females paired with horned males construct larger brood masses than those paired with hornless males or breeding alone (Hunt and Simmons 1998), suggesting ASR may shape offspring development via altered parental investment. However, it remains unresolved whether ASR effects on offspring arise from independent action or interaction between these two TGP pathways.

To dissect the respective contributions of the two core pathways underpinning ASR‐induced TGP, we developed an egg‐transplantation protocol in *O. taurus* . We transferred eggs from their original brood balls, which vary in mass reflecting parental investment, into standardized artificial brood balls. Assessing offspring development within this common gestational environment isolates germline‐mediated TGP, while the original brood ball mass directly quantifies parental investment. We hypothesize parental ASR influences offspring development through two non‐mutually exclusive pathways: (1) Germline‐mediated TGP: ASR‐induced stress drives nongenetic developmental programming. To adapt to intense competition, offspring from male‐biased populations will exhibit accelerated developmental speed and reduced developmental duration. (2) Parental investment‐mediated TGP: ASR alters nutritional provisioning. In male‐biased contexts, males may increase brood ball provisioning to secure paternity, producing heavier brood balls and yielding larger offspring.

## 2 Materials and Methods

### 2.1 Establishment of the Laboratory Colony

The laboratory colony of *Onthophagus taurus* was established from 100 founders (50 males, 50 females) collected in March 2018 from the Terrestrial Ecotoxicology Laboratory (Niefern‐Öschelbronn, Germany) and transported to the University of Groningen (Groningen, Netherlands). Beetles were shipped over 2 days via ground courier in plastic buckets (30 × 40 cm, diameter × height) containing moist sand without dung, sealed with plastic film to minimize air exchange. Upon arrival, the containers were opened in a climate‐controlled room (22°C ± 3°C, 16:8 h L:D photoperiod). No mortality was observed. While we acknowledge that zero mortality is a crude metric that cannot capture sublethal physiological stress, all founder beetles underwent a minimum of 6 weeks of acclimatization under optimal, standardized laboratory conditions prior to any breeding. This extended period ensures that any transient physiological effects of transport would have fully dissipated, and all individuals entered the experiment with a uniform physiological baseline. Although mating activity during transit could not be excluded, spontaneous oviposition was observed in a subset of females shortly after arrival. To ensure genetic control, these eggs were euthanized by freezing at −20°C to prevent the inclusion of offspring with unknown paternity in the stock colony.

Founders were housed individually in boxes (10 × 7 × 8 cm, Length (L) × Width (W) × Height (H)) within the climate‐controlled room on a 1:1 soil‐sand substrate. To maintain optimal and uniform physiological and reproductive status, beetles were provisioned with 60 g of fresh dung every 5 days during the 6‐week acclimatization. To produce subsequent generations, each male–female pair cohabited in an individual breeding chamber (16 × 9 × 10 cm, L × W × H) filled three‐quarters with a moist soil‐sand mixture and topped with 200 g of fresh dung. After a 5‐day oviposition period in the presence of the male, completed brood balls were collected, cleaned, and individually reburied until emergence. Emerging adults were subsequently allocated for experiments or used to maintain the colony following this standardized pairwise breeding protocol.

### 2.2 Maintenance of Experimental Parents

Virgin adults from the third laboratory generation were used for all experiments. Upon emergence, these beetles were maintained under the aforementioned housing, feeding, and environmental conditions. Each beetle was used only once. All selected individuals were sexually mature, morphologically intact, vigorous, and free of visible parasites. Fresh dung from an organic, pesticide‐free dairy farm in northern Groningen (Martinizicht, 53°15′37.8″N, 6°34′45.5″ E) was stored at −20°C in 10‐L buckets. Prior to feeding or experimental use, the dung was thawed at room temperature for 24 h and thoroughly homogenized. Further methodological details are described in Zhang et al. (2024).

### 2.3 Transplantation System

#### 2.3.1 Basic Structure of Artificial Brood Balls

**FIGURE 1:**
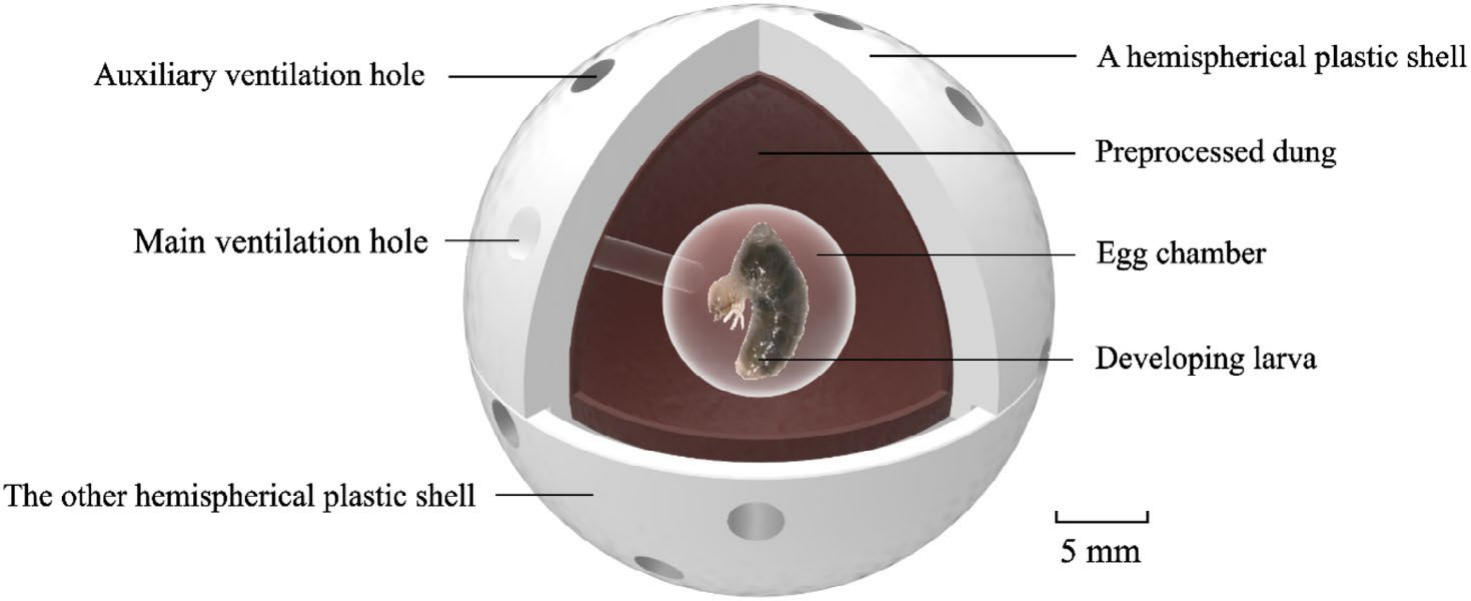
Internal architecture of a biomimetic artificial brood ball for dung beetles. This three‐dimensional perspective view illustrates the nested structure of the artificial brood ball. The outer casing comprises two hemispherical plastic shells that enclose a matrix of preprocessed dung. A central egg chamber, shown here with a developing larva, mimics the microenvironment of a natural brood ball. Integrated main and auxiliary ventilation holes ensure continuous air exchange and oxygen supply for the developing offspring. This design enables noninvasive, in situ monitoring of larval development throughout metamorphosis. Photographs of the assembled prototype are shown in Figure 3d,e.

Artificial brood balls were fabricated by modifying traditional Chinese herbal pill casings (GB 13731, China), which were purchased online from a commercial supplier (Taobao, China). Each casing comprises two detachable, interlocking hemispherical shells forming a complete hollow sphere with an internal diameter of ~30 mm. To ensure adequate oxygen diffusion for offspring development, six ventilation holes (~2 mm diameter) were evenly drilled into each hemisphere using a mini electric drill (DZTZ02, Gerbala), yielding 12 holes per ball. Additionally, 15 g of preprocessed dung was placed inside to serve as food throughout larval development (Figures 1 and 3d,e). This standardized internal space and food quantity fully supported development, ensuring each larva experienced a strictly consistent and uniform nutritional environment.

#### 2.3.2 Preparation and Preloading of Preprocessed Dung

Preprocessed dung was prepared by mixing fresh and dried dung to approximate natural moisture levels. Fresh dung was thawed for 24 h and equilibrated to room temperature. Dried dung was produced by spreading fresh dung (< 1 cm thick) onto acrylic plates, oven‐drying at 60°C for 48 h to a constant weight, and grinding it into a fine powder. During formulation, fresh dung was placed in a stainless‐steel bowl (20 cm diameter, 2 L capacity), and the dried powder was gradually incorporated. The mixture was continuously stirred with steel tweezers until visual and tactile evaluations confirmed a homogeneous consistency resembling natural brood balls. Humidity levels in the artificial brood balls were maintained to approximate natural conditions by controlling the fresh‐to‐dried dung ratio. Suitability of the dung mixture was assessed through visual and tactile evaluation.

The preprocessed dung was firmly packed into each hemispherical shell. A central cavity was then excavated using forceps, and its surface was smoothed and compressed with blunt‐tipped forceps. When joined, the hemispheres formed a central chamber (~1 cm diameter) for egg transplantation. A fine sewing needle (0.5 mm in diameter) was then inserted through one ventilation hole into the cavity to establish an air channel for connecting the cavity to the external environment. Alongside the auxiliary holes, this facilitated air exchange simulating natural developmental conditions (Figure 1). During experiments, this channel's exterior was sealed with soil to block light and restrict airflow. On the morning of transplantation, ~250 artificial brood balls were prepared and stored statically in a large container, ensuring the dung substrate and internal air fully equilibrated to ambient room temperature before use.

#### 2.3.3 Transferring Fertilized Eggs From Original Brood Balls to Artificial Brood Balls

During transplantation, the dung plug at each original brood ball's aperture was dislodged using fine‐pointed forceps. After clearing surrounding debris, the ball was opened to expose its contents (Figure 3a). Dislodged dung fragments were subsequently recompacted into the original brood balls to prevent confounding parental care measurements. Using 75% ethanol‐sterilized blunt‐tipped forceps, gentle pressure was applied to the egg's mid‐region to detach and transfer it into the artificial brood ball's cavity. All procedures were performed with gloves to prevent mechanical damage and microbial contamination. Inside the artificial cavity, the egg's basal region adhered to the substrate while the remainder remained air‐exposed. This methodology was developed drawing inspiration from the foundational larval rearing frameworks described in Emlen and Nijhout (2001) and Moczek and Nijhout (2002). It is designed to simulate the core physical structural features of natural brood balls, including the central egg chamber and gas exchange pathways, under controlled artificial conditions, ensuring that the developmental environment closely approximates that experienced by eggs in their natural subterranean habitat.

#### 2.3.4 Assessment of the Transplantation System

To evaluate system feasibility, a pilot experiment was conducted 1 week before the main study. Ten male–female pairs were housed individually in breeding boxes (16 × 9 × 10 cm, L × W × H) containing 100 g of fresh dung atop soil. After a 3‐day breeding period, 37 naturally produced brood balls were collected. Eggs from these balls were individually transplanted into artificial brood balls using the described protocol. Following a 5‐day incubation, all artificial balls were opened, revealing successful larval development in 35 of 37 attempts (94.59% success rate). These findings demonstrate that the designed transplantation system is safe, efficient, scalable, and highly practical.

### 2.4 Experimental Procedure

#### 2.4.1 Alignment of ASR and OSR in the Experimental System

Although the demographic adult sex ratio (ASR) conceptually differs from the operational sex ratio (OSR), the ratio of sexually active males to receptive females (Emlen and Oring 1977), we consider both metrics functionally equivalent within our experimental framework. In nature, OSR fluctuates independently of ASR. However, our design exclusively utilized sexually mature individuals in peak physiological condition, ensuring continuous mating capability and receptivity. Furthermore, conducting trials in spatially confined arenas maximized interindividual encounter rates and prevented temporary withdrawal from the mating pool. Under these controlled conditions, the manipulated ASR directly dictated immediate competitive pressure, generating distinct and consistent gradients of OSR and socially mediated stress across treatments.

#### 2.4.2 ASR Treatments Design

The experiment was conducted from November to December 2021. A total of 180 adults were randomly assigned to three ASR treatments: female‐biased (FB), unbiased (UB), and male‐biased (MB) (Table 1). Previous results indicated that ASR did not significantly affect parental investment, measured as dried brood ball weight, under a narrower range (FB: 2 M:4F, 33%; UB: 3 M:3F, 50%; MB: 4 M:2F, 66%, Zhang et al. 2024). To elucidate ASR effects more clearly and achieve a steeper gradient under a fixed total sample size, we varied the replicate group sizes. Specifically, unbiased replicates contained 4 individuals (UB: 2 M:2F, 50%), whereas biased replicates contained 8 individuals (FB: 2 M:6F, 25%; MB: 6 M:2F, 75%). To equalize population density, experimental arena sizes were scaled proportionally to group size, keeping per capita space constant (details below). We acknowledge that despite standardized density, group size itself may independently influence competitive interactions, mating dynamics, and TGP effects. To mitigate this, our core between‐treatment comparisons focused predominantly on the FB and MB groups. Because these two treatments were fully matched for group size, density, and all other non‐focal variables, this approach eliminated the group size confounder, providing a robust framework for hypothesis testing. The UB treatment was fully included in statistical models as a neutral baseline to contextualize ASR effects. Furthermore, this design ensured baseline intrasexual competition (≥ 2 same‐sex individuals per replicate) across all treatments.

**TABLE 1:** Experimental setup for adult sex ratio (ASR) manipulation.

Treatment	ASR (%)	Replication	Number of males	Number of females	Body size of males	Body size of females
Female‐biased	25	× 9	2	6	5.015 ± 0.062	4.898 ± 0.031
Unbiased	50	× 9	2	2	4.958 ± 0.040	4.927 ± 0.055
Male‐biased	75	× 9	6	2	4.857 ± 0.043	4.950 ± 0.077

*Note:* Body size is given as mean ± SE.

**FIGURE 2:**
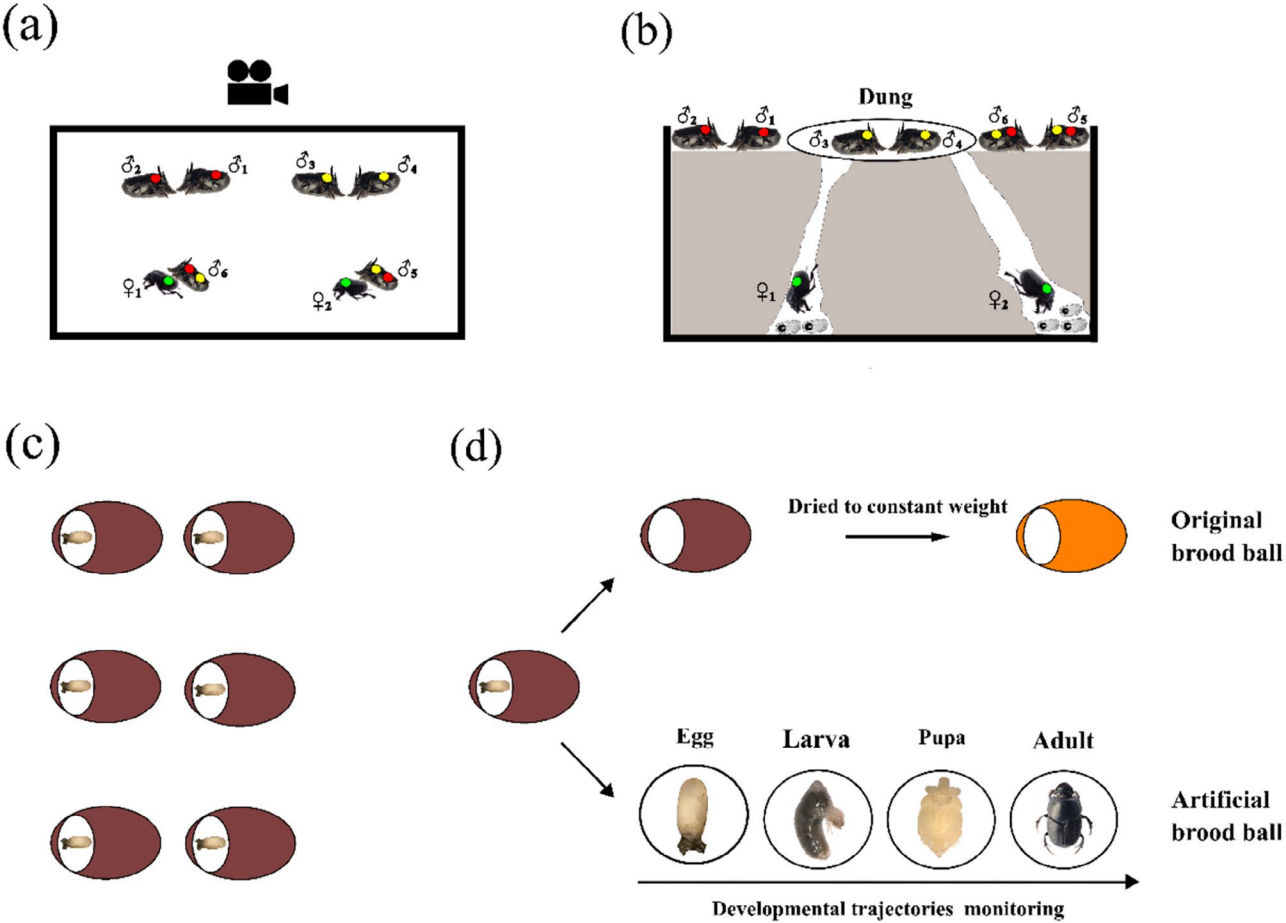
Schematic overview of the full experimental protocol, with the male‐biased (MB) treatment shown as a representative example. All three ASR treatments followed an identical workflow, proceeding in sequence from (a) to (d). (a) Pre‐breeding behavioral observation. Beetles were individually marked (males: Red/yellow; females: Green) and video‐recorded for 90 min to quantify contest and courtship interactions. (b) Breeding phase. Parental beetles were transferred to standardized breeding chambers for a 5‐day nesting period. (c) Brood ball collection. Brood balls were harvested and cleaned of adhering soil and debris. (d) Sample processing and offspring monitoring. The workflow bifurcated: Original brood balls were dried to constant weight to measure parental investment (upper path), while eggs were extracted from original brood balls and transplanted into standardized artificial brood balls to track offspring developmental trajectories (lower path). Drawings of developmental stages are adapted from Jones et al. (2025).

All beetles mated freely within their assigned replicate populations for the entire breeding period, ensuring individuals experienced the full social context of the manipulated ASR, including unconstrained mating interactions and intrasexual competition dynamics. *O. taurus* is an obligately polyandrous species characterized by intense pre‐ and postcopulatory sexual selection and sperm competition (McCullough et al. 2017; Simmons and Holley 2011). This mating system can alter the genetic composition of offspring, consequently shaping their developmental trajectories during metamorphosis. A standard protocol to control offspring genetic background involves mating females exclusively with males from the same full‐sib family line (Sato et al. 2023). However, following our established rearing protocol, all experimental males were derived from random mating in the preceding generation, precluding the definitive characterization of their individual genetic backgrounds. To address this limitation, we applied a fully randomized assignment strategy for all parental beetles across treatments. This randomization homogenizes sire genetic variance across ASR groups, effectively mitigating potential biases driven by intense sexual selection or sperm competition. All replicates within each treatment ran simultaneously. For identification, beetles were individually marked with colored dots on the pronotum and elytra (Edding 751, Japan) without impairing movement (Figure 2a). Pronotum length, measured via a digital caliper (±0.01 mm, Mahr, Germany), proxied body size. Parental body size did not differ significantly between sexes or among ASR treatments (Sex: *F* = 0.076, DF = 1, *p* = 0.784; ASR treatment: F = 0.918, DF = 2, *p* = 0.401) (Table 1).

#### 2.4.3 Pre‐Breeding Behavioral Observation

To ensure individuals experienced corresponding ASR‐induced stimuli, beetles from each group were monitored in treatment‐specific boxes. We investigated the impact of ASR on competition intensity, a primary source of social pressure, by quantifying the frequencies of contest and courtship behaviors (Figure 2a). To maintain consistent density across treatments, transparent plastic box dimensions varied: 16 × 9 × 10 cm (L × W × H, ~17 cm^2^/ind) for biased treatments and 10 × 7 × 8 cm (~18 cm^2^/ind) for the control. The inner surface of each box was covered with a thin layer of soil that did not obstruct behavioral observations. Crucially, while this shallow soil depth allowed individuals to non‐obstructively engage in courtship and contest behaviors, it prevented them from successfully completing mating. Three boxes, representing each ASR treatment, were spaced 50 cm apart under uniform dim lighting at 22°C ± 3°C. An overhead video camera (JVC, GZ‐R405BEU, Japan) recorded individual movements for 90 min (Figure 2a). This specific duration ensured adequate exposure to ASR‐induced stress while simultaneously minimizing the risk of physical injury.

During pre‐breeding observations, definitions and quantitative protocols for contest and courtship behaviors followed Zhang et al. (2024). Briefly, contest behavior involved agonistic physical combat, predominantly head‐butting and pushing (Beckers et al. 2017). Although primarily occurring between males, contests involving females or intersexual interactions were also observed (Figure 2a). We quantified total contest events per box to evaluate ASR impacts on contest intensity. Courtship behavior was characterized by a male repeatedly drumming its foreleg tarsi on a female's pronotum or elytra (Figure 2a, Beckers et al. 2017). We recorded total courtship initiations per box to assess ASR effects on courtship intensity.

#### 2.4.4 Breeding Protocol and Brood Balls Collection

**FIGURE 3:**
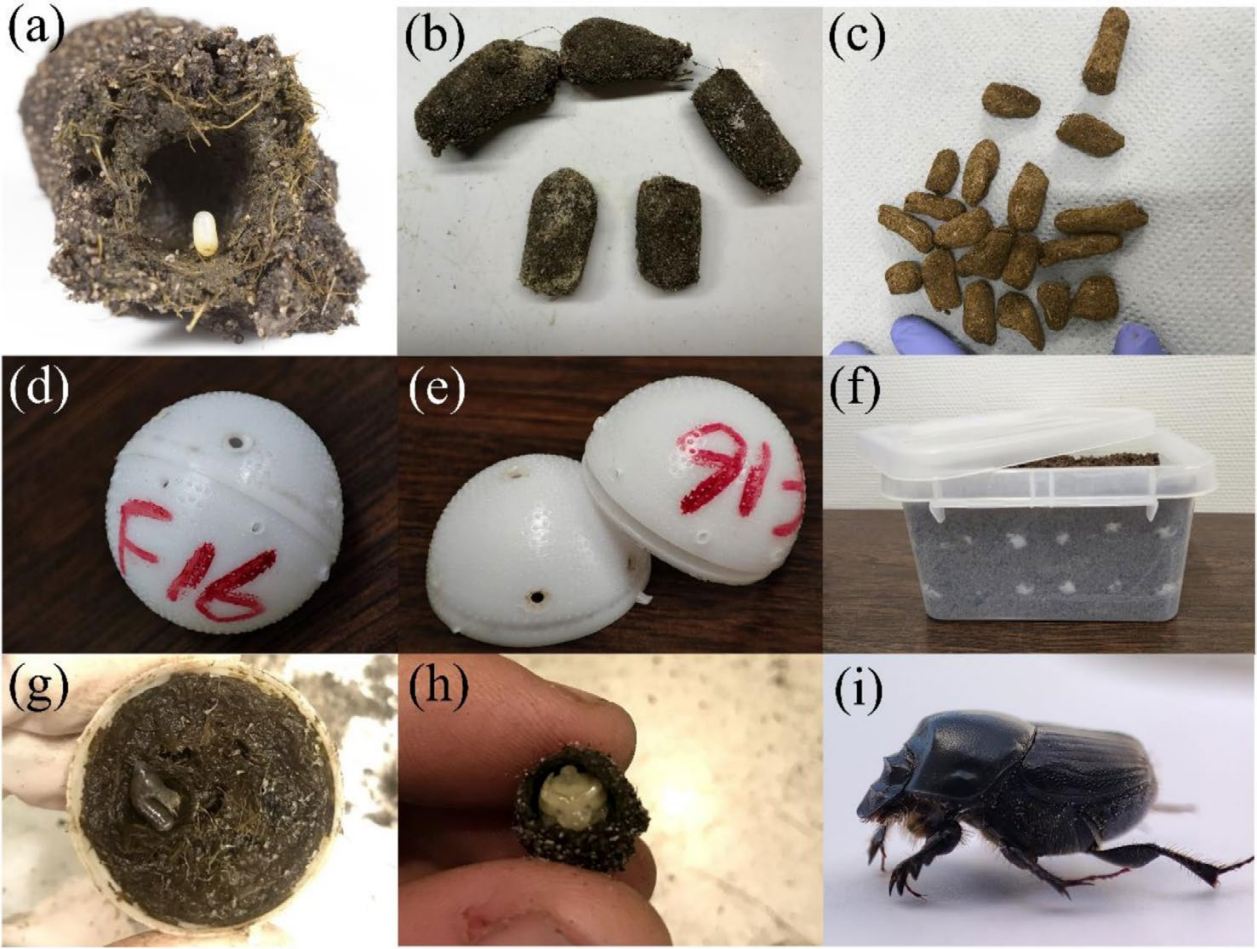
Photographic documentation of the experimental components and offspring developmental stages. (a) Cross‐section of a natural brood ball, showing a fertilized egg in the central oviposition chamber. (b) Intact natural brood balls after removal of adhering soil. (c) Natural brood balls dried to constant mass for quantification of parental investment. (d, e) Assembled artificial brood ball system (see Figure 1 for schematic), consisting of ventilated plastic hemispheres enclosing standardized preprocessed dung. (f) Incubation setup, with artificial brood balls buried in a moist soil–sand substrate under controlled environmental conditions. (g–i) Offspring developmental stages monitored within the artificial system: (g) feeding larva, (h) pupa, and (i) newly emerged adult *O. taurus* . Photo credit: Panel (a) from Rohner et al. (2024); panels (b–i) by Lisheng Zhang.

Post‐observation, all individuals were transferred to breeding chambers structurally identical to their corresponding observation boxes. These chambers were filled three‐quarters with a 1:1 moist sand‐soil mixture and topped with fresh dung (200 g for FB and MB; 100 g for UB) for breeding (Figure 2b). After a 5‐day breeding period, brood balls were extracted and sieved through a 0.5‐cm mesh (Figure 2c, Figure 3b). Adhering soil was meticulously removed with fine forceps, and internal fecal impurities were extracted using a micro‐dissecting needle (Hunt and Simmons 2002a).

### 2.5 Assessment of Offspring Development

#### 2.5.1 Somatic Growth

Intact brood balls were dissected (as detailed above) to separate the eggs from the remaining dung mass. For developmental assessment, these eggs were individually transplanted from their original brood balls into artificial brood balls following the established protocol. Each artificial brood ball was externally marked for identification using a permanent marker (Edding 751, Japan). Subsequently, artificial brood balls across all ASR treatments were randomly buried in soil identical to that of the breeding chambers (Figure 3f, temperature: 20°C ± 3°C; volumetric water content: ~28%–35%, measured via a WET‐2 sensor, Delta‐T Devices Ltd., UK).

To assess larval developmental stage and body mass, a noninvasive weighing procedure was performed at regular intervals. During each inspection, buried artificial brood balls were gently excavated, and surface soil was removed using a soft brush. Each ball was carefully halved along its interlocking seam using a utility knife, and the developing larva was extracted with blunt‐tipped forceps. Prior to weighing, adhering dung fragments were carefully removed; occasional excretions of dark ruminated fluid during handling were not accounted for in the body mass. Larvae were weighed on an electronic balance (±0.0001 g, Mettler Toledo, AG204, Switzerland), using a fresh sheet of white paper for each individual to strictly prevent cross‐contamination. After weighing, each larva was returned to its original position within the artificial brood chamber (Figure 3g–i). To prevent adverse developmental effects, all procedures were conducted at ambient room temperature under dim lighting to avoid direct light exposure. To ensure measurement consistency, weighing sessions were strictly completed within a 3‐h window. To satisfy this logistical constraint, we subsampled the female‐biased treatment, selecting 60 of 107 artificial brood balls for developmental assessment. In contrast, because brood ball production in the unbiased and male‐biased treatments was inherently limited, all available fertilized eggs from these groups were transplanted to permit comprehensive developmental tracking.

#### 2.5.2 Developmental Stages Testing

At each sampling point, offspring developmental stages were recorded to calculate the durations of the larval, pupal, and emerging phases. The larval stage spanned from hatching to pupation, characterized by mobility and active feeding using mandibular mouthparts (Figure 3g). The pupal stage extended from pupation to adult emergence (Figure 3h), during which individuals ceased feeding, became immobile, and underwent significant morphological metamorphosis to resemble the adult form. Adult emergence was defined as the point when newborn beetles could freely stretch their limbs and move (Figure 3i, Hanski and Cambefort 2014; Scholtz et al. 2009). Because the precise timing of original brood ball production was untrackable, egg stage duration was excluded from our analyses. We recorded offspring peak weight and the time required to reach it. Developmental speed, an indicator of growth rate, was calculated as the ratio of peak weight to this developmental time (g/days). Upon adult emergence, we recorded offspring sex, emerging weight, body size, and male horn size. Because emerging weight exhibited an extremely positive correlation with both body size (Pearson correlation, *r* = 0.902, *p* < 0.001) and male horn size (*r* = 0.873, *p* < 0.001), we retained only the emerging weight variable in our final results analyses to prevent redundancy.

#### 2.5.3 Measurement of Parental Care

Posttransplantation, remaining eggless original brood balls were dried to a constant weight at 60°C for 48 h. Following adherent sand removal via micro‐probes, masses were measured on an electronic balance (±0.0001 g, Mettler Toledo, AG204, Switzerland) (Figure 3c) to proxy parental investment.

### 2.6 Statistical Analyses

All statistical analyses were conducted in R v4.3.1 (R Core Team 2023). Linear mixed‐effects models (LMMs) were fitted using the “lme4” package (Bates et al. 2015), with fixed‐effect *p*‐values estimated via “lmerTest” (Kuznetsova et al. 2017). Residual normality and homoscedasticity were verified using simulation‐based diagnostics in “DHARMa” (Hartig 2022). Initial models included ASR treatment, sex, and relevant interactions as fixed effects. Following Akaike Information Criterion (AIC) model selection, nonsignificant interactions were iteratively removed, retaining all main effects. To account for hierarchical data structure, experimental group (box) and/or offspring ID were included as random intercepts. Post hoc pairwise comparisons with multiple testing adjustments were performed using the “emmeans” (Lenth 2023) and “multcomp” packages (Hothorn et al. 2008).

#### 2.6.1 Intensity of Contest and Courtship Behavior

We analyzed contest and courtship frequencies using an LMM with ASR treatment, behavior type, and their interaction as fixed factors. Experimental group was included as a random intercept to account for within‐group nonindependence.

#### 2.6.2 Assessment of Offspring's Development

Paired *t*‐tests initially examined differences between developmental stages. To assess ASR treatment and sex effects on developmental speed and emergence weight, separate LMMs were fitted. Because ASR × sex interactions were nonsignificant for both variables, they were excluded, retaining only main effects in the final models. Experimental group was included as a random intercept. To analyze stage‐specific developmental durations, data were structured longitudinally and analyzed via an LMM including ASR treatment, sex, and developmental stage as fixed factors (Snijders and Bosker 2011). Following AIC‐based evaluation of two‐ and three‐way interactions, the full three‐way interaction model provided the best fit and was retained. Experimental group and individual ID were included as random intercepts to account for repeated measures across stages.

#### 2.6.3 Parental Care Testing

Parental care, proxied by dried brood ball mass, was compared across ASR treatments using an LMM. ASR treatment was specified as the fixed effect, with experimental group included as a random intercept.

## 3 Results

### 3.1 Intensity of Contest and Courtship Behaviors Among Different ASR Treatments

**FIGURE 4:**
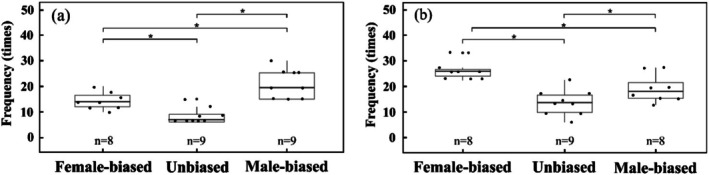
Effect of adult sex ratio (ASR) on the frequency of (a) contest and (b) courtship behaviors. The plots show the total number of behavioral events recorded per replicate arena over 90 min observation period. Box plots indicate the median (central thick line), interquartile range (box boundaries), and data range within 1.5 × IQR (whiskers). Black dots represent individual replicates. Sample sizes (*n*) are shown above the *x*‐axis for each treatment (FB, Female‐biased; MB, Male‐biased; UB, Unbiased). Post hoc pairwise comparisons were performed with Tukey's HSD correction for multiple comparisons; asterisks indicate significant differences between treatments at *p* < 0.05.

The effects of ASR on these behaviors differed significantly, as evidenced by a significant interaction effect between ASR treatments and behavior types (Table 2). For contest behavior, the frequency of contests in the male‐biased treatment was higher compared to both unbiased (*t* = 5.956, *p* < 0.001) and female‐biased treatments (*t* = 2.990, *p* = 0.014), while the frequency of contests in the female‐biased treatment surpassed that of the unbiased treatment (*t* = 2.789, *p* = 0.023) (Figure 4a). For courtship behavior, both male‐biased (*t* = 2.767, *p* = 0.025) and female‐biased (*t* = 5.759, *p* < 0.001) treatments exhibited significantly higher frequency than the unbiased treatment; additionally, the courtship frequency in female‐biased treatment was significantly higher than that in male‐biased treatment (*t* = 2.971, *p* = 0.015) (Figure 4b). These results confirm that our ASR manipulations successfully induced the predicted gradients of social stress and behavioral plasticity in parental beetles, validating the experimental premise for testing transgenerational effects.

**TABLE 2:** The influence of adult sex ratio (ASR) on intensity of contest and courtship behavior in dung beetle *Onthophagus taurus* during a 90 min trial.

Explanatory variable	Mean ± SE	*F*	DF	*p*
Intensity of contest and courtship behaviors				
ASR treatment		14.996	2	**< 0.001**
Behavior types		16.928	1	**< 0.001**
ASR treatment: Behavior types		24.213	2	**< 0.001**
Contest frequency in each box (times)				
Female‐biased (*n* = 8)	14.500 ± 1.180			
Unbiased (*n* = 9)	8.333 ± 1.067			
Male‐biased (*n* = 9)	21.111 ± 1.866			
Courtship frequency in each box (times)				
Female‐biased (*n* = 8)	26.625 ± 1.223			
Unbiased (*n* = 9)	13.888 ± 1.719			
Male‐biased (*n* = 8)	19.625 ± 2.008			

*Note:* Data are presented as mean ± SE per observation box. A LMM was fitted with ASR treatment and behavior type as fixed main effects, including their interaction term. Observation box identity was included as a random factor. Bold text indicates significant differences (*p* < 0.05).

### 3.2 Larval Development

**FIGURE 5:**
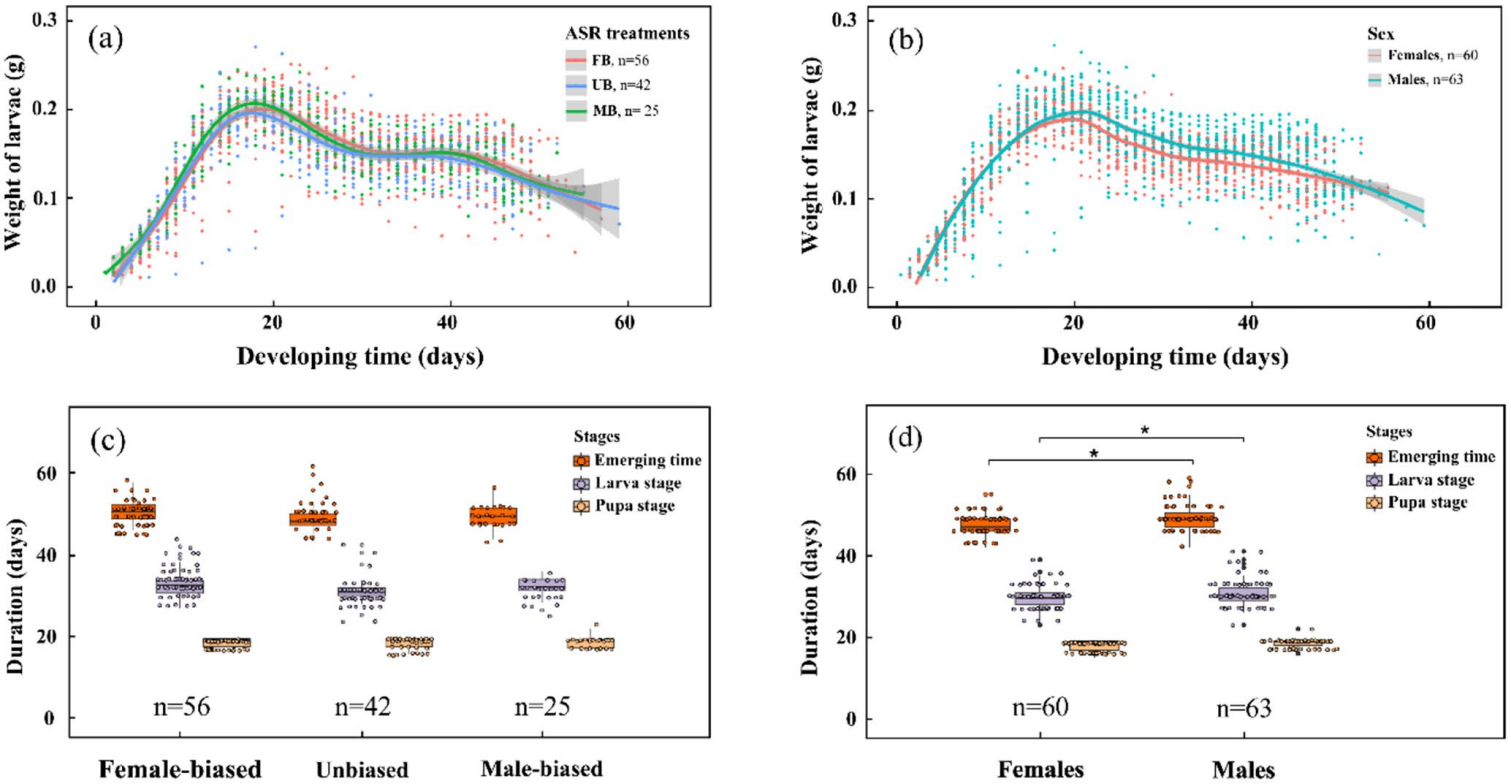
Offspring growth trajectories and developmental duration across adult sex ratio (ASR) treatments and sexes. (a, b) Changes in offspring body weight (measured every 48 h) throughout development. Each point represents the raw measured value from a single individually tracked offspring at each 48‐h sampling interval. Curves represent smoothed growth trends with 95% confidence intervals (shaded areas), compared among (a) ASR treatments (FB, Female‐biased; MB, Male‐biased; UB, Unbiased) and (b) offspring sexes. (c, d) Duration of larval, pupal, and total emergence periods compared among (c) ASR treatments and (d) offspring sexes. Color coding of points corresponds to distinct developmental stages across all individual offspring. Box plots indicate the median (thick central line), interquartile range (IQR, box boundaries), and data range within 1.5 × IQR (whiskers); Sample sizes (*n*) are indicated within each panel. Linear mixed‐effects models were used to test for treatment effects; asterisks denote significant differences between groups at *p* < 0.05.

A total of 60 fully developed females (24 in FB, 23 in UB, and 13 in MB) and 63 males (32 in FB, 19 in UB, and 12 in MB) offspring were collected. ASR had no effect on larval development, with no significant differences observed in development speed among the three treatments and sexes (Table 3; Figure 5a,b), A detailed visualization of the growth trajectories separated by both ASR treatment and offspring sex confirms this consistent pattern (Appendices, Figure S1). In addition, no significant differences in emerging weight were detected among treatments and sexes (Table 3). This finding directly contradicts our first hypothesis, providing no evidence for germline‐mediated transgenerational plasticity in offspring growth traits in response to parental ASR experience.

**TABLE 3:** The influence of parental adult sex ratio (ASR) on the somatic development of male and female larvae in dung beetles *Onthophagus taurus* .

Explanatory variables	Mean ± SE	*F*	DF	*p*
Developing speed (g/day)				
ASR treatment		0.859	2	0.446
Sex		0.025	1	0.874
ASR treatment: Sex		1.713	2	0.185
Female‐biased (*n* = 56)	0.010 ± 0.001			
Unbiased (*n* = 42)	0.012 ± 0.001			
Male‐biased (*n* = 25)	0.011 ± 0.001			
Males (*n* = 63)	0.011 ± 0.001			
Females (*n* = 60)	0.011 ± 0.001			
Emerging weight (g)				
ASR treatment		0.115	2	0.891
Sex		0.221	1	0.639
ASR treatment: Sex		0.789	2	0.456
Female‐biased (*n* = 56)	0.102 ± 0.002			
Unbiased (*n* = 42)	0.102 ± 0.002			
Male‐biased (*n* = 25)	0.103 ± 0.002			
Males (*n* = 63)	0.102 ± 0.002			
Females (*n* = 60)	0.103 ± 0.002			

*Note:* Data are presented as mean ± SE. Separate LMMs were fitted for developing speed and emerging weight. ASR treatment and sex were included as fixed main effects. The interaction term (ASR treatment × sex) was nonsignificant and removed from the final models. Observation box identity was included as a random factor.

Total developmental time until emergence comprised two distinct phases: the larval stage and the pupal stage. Specifically, larval duration was significantly longer than pupal duration (paired *t*‐test: *t* = 33.80, *p* < 0.001, mean difference = 12.07 days). In contrast, total time to emergence was significantly longer than both larval duration (*t* = −155.13, *p* < 0.001, mean difference = −18.18 days) and pupal duration (*t* = −103.28, *p* < 0.001, mean difference = −30.24 days). These results indicate that the larval phase contributed the majority of the overall developmental period, while the pupal stage was considerably shorter (Figure 5c,d). Consistent with the growth results, ASR was not a significant predictor of developmental stage duration (Table 4). Although no significant interaction was observed between sex and developmental stage in our model, post hoc analyses revealed that males exhibited longer durations in both the larval (*t* = 2.596, *p* = 0.009) and total emergence time (*t* = 3.625, *p* < 0.001) stages compared to females; no significant sex difference was detected in pupal duration (*t* = 1.192, *p* = 0.234) (Table 4; Figure 5c,d). A detailed breakdown confirms that these sex‐specific differences in developmental duration remained consistent across all three ASR treatments (Appendixes, Figure S2). These stage‐specific analyses further reinforce our core finding that parental ASR experience does not alter offspring developmental trajectories, even when examined at finer temporal resolution.

**TABLE 4:** The influence of parental adult sex ratio (ASR) on the duration of developmental stages in male and female larvae in dung beetles *Onthophagus taurus* .

Explanatory variables	Mean ± SE	F	DF	*p*
Duration of development (days)				
ASR treatment		0.388	2	0.683
Sex		18.030	1	**< 0.001**
Stages		5340.096	2	**< 0.001**
ASR treatment: Stages		1.0760	4	0.229
Sex: Stages		0.432	2	0.095
ASR treatment: Sex: Stages		1.893	4	0.112
Duration of larva stage (days)				
Female‐biased (*n* = 56)	30.964 ± 0.450			
Unbiased (*n* = 42)	29.500 ± 0.520			
Male‐biased (*n* = 25)	29.880 ± 0.480			
Males (*n* = 63)	30.951 ± 0.433			
Females (*n* = 60)	29.524 ± 0.374			
Duration of pupa stage (days)				
Female‐biased (*n* = 56)	18.196 ± 0.165			
Unbiased (*n* = 42)	18.142 ± 0.200			
Male‐biased (*n* = 25)	18.200 ± 0.294			
Males (*n* = 63)	18.370 ± 0.156			
Females (*n* = 60)	17.893 ± 0.172			
Duration of emerging time (days)				
Female‐biased (*n* = 56)	49.162 ± 0.370			
Unbiased (*n* = 42)	47.642 ± 0.490			
Male‐biased (*n* = 25)	48.080 ± 0.547			
Males (*n* = 63)	49.322 ± 0.382			
Females (*n* = 60)	47.508 ± 0.338			

*Note:* Data are presented as mean ± SE. A LMM was fitted with ASR treatment, sex, and developmental stage as fixed main effects, including all two‐way and three‐way interaction terms. Observation box identity and individual identity were included as random factors to account for repeated measures across developmental stages. Bold text indicates significant differences (*p* < 0.05).

### 3.3 Parental Care

**FIGURE 6:**
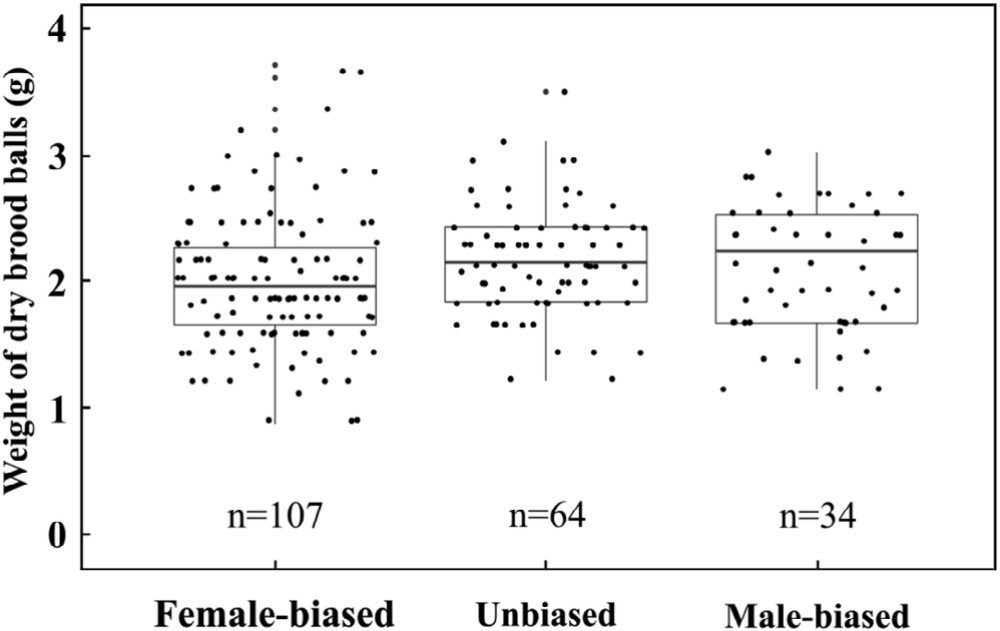
Effect of adult sex ratio (ASR) on parental investment. The plot illustrates the dry mass of natural brood balls across three ASR treatments (FB, Female‐biased; MB, Male‐biased; UB, Unbiased). Each point represents the weight of an individual brood ball, measured after being dried to a constant mass following egg extraction. Box plots depict the median (thick horizontal line), 25th and 75th percentiles (box boundaries), and data range within 1.5 × the interquartile range (whiskers). Sample sizes (*n*) for each treatment are indicated above the x‐axis. A linear mixed‐effects model was used to test for the effect of ASR treatment on brood ball mass, with replicate arena included as a random intercept.

A total of 205 dried to constant weight brood balls were collected (107 in FB, 64 in UB, and 34 in MB). ASR had no impact on parental care, as evidenced by the lack of significant differences in dried brood ball weight among the three treatments (Table 5; Figure 6). This result rejects our second hypothesis, indicating that ASR does not influence offspring development via altered post‐zygotic parental provisioning of brood balls.

**TABLE 5:** The influence of adult sex ratio (ASR) on parental care (measured as brood ball mass) in the dung beetles *Onthophagus taurus* .

Explanatory variable	Mean ± SE	*F*	DF	*p*
Weight of dried brood balls in each box (g)				
ASR treatment		0.851	2	0.445
Female‐biased (*n* = 107)	1.992 ± 0.052			
Unbiased (*n* = 64)	2.137 ± 0.057			
Male‐biased (*n* = 34)	2.109 ± 0.088			

*Note:* Data are presented as mean ± SE. A linear mixed‐effects model (LMM) was fitted with ASR treatment as the fixed main effect. Observation box identity was included as a random factor to account for nonindependence within groups.

## 4 Discussion

Our study successfully disentangled the two core nongenetic TGP pathways mediating ASR effects: behavioral parental investment and germline‐mediated transmission. Our results yield two key conclusions. First, ASR is a potent driver of parental behavioral plasticity: male‐biased ASR intensified contest competition, while female‐biased ASR maximized courtship intensity. Second, despite these profound behavioral adjustments and associated social stress, we found no evidence of transgenerational transmission via either pathway. Offspring developmental trajectories remained robust to parental social environment under standardized rearing conditions, suggesting that while *O. taurus* adults are highly sensitive to social demography, offspring development is canalized against such fluctuations, potentially via the buffering brood ball system.

### 4.1 ASR'S Influence on Contest and Courtship Behavior in Parents

Our results demonstrate that ASR significantly influenced parental contest and courtship dynamics. Contest frequency peaked in the male‐biased treatment and was also elevated in the female‐biased treatment compared to the unbiased control. While the male‐biased maximum is consistent with our previous work (Zhang et al. 2024), the elevated competition under female‐bias is a novel finding that partially aligns with the nonlinear OSR‐contest relationship reported by Weir et al. (2011). For courtship, frequencies were significantly lower in the unbiased control than in either biased treatment. However, a key divergence emerged: while Zhang et al. (2024) reported peak courtship under male‐biased conditions, courtship in this study peaked in the female‐biased treatment. This partially corresponds with Weir et al. (2011), who observed decreased courtship as ASR male‐bias increased. These discrepancies suggest that when female availability is high and male interference is low, males may shift strategies from competitive scrambling to enhanced individual courtship. This strategic reallocation of reproductive effort highlights that ASR‐mediated behavioral modulation is a complex trade‐off influenced by social environment and species‐specific traits, rather than a simple linear response to competition. These shifts conform to sexual selection theory; as noted by Székely et al. (2014), male‐biased environments intensify competition, whereas female‐biased conditions predict reduced aggression and increased courtship (Clark and Grant 2010; Forsgren et al. 2004; Grant et al. 2000). Ultimately, these variations reflect time and energy trade‐offs dictated by mate encounter rates (Grant et al. 2000). Collectively, biased ASRs subjected individuals to intense, continuous social pressures. Crucially, these interactions provided pre‐mating perceptual experience of the social environment rather than actual sexual experience, which in dung beetles necessitates copulation (Chamorro‐Florescano and Favila 2008).

### 4.2 ASR'S Influence on the Development of Offspring

Parental experience shapes offspring phenotype and fitness via nongenetic TGP. This phenomenon is widespread in insects, where rapid generation times and overlapping parental‐offspring environments favor sophisticated transgenerational signaling (Bell and Hellmann 2019; Bonduriansky 2021). Adaptive TGP is triggered by diverse social cues: high density induces winged dispersal in aphids (Braendle et al. 2006), while heightened male–male competition produces crickets with more aggressive courtship (Noguera 2023). Crucially, in *O. taurus* , maternal exposure to high density induces transgenerational weapon enlargement in major males (Buzatto et al. 2012). Because this effect occurred independently of brood ball mass, it indicates transmission via non‐nutritional mechanisms, such as egg composition or maternal brood modifications. Because skewed ASRs alter intra‐ and intersexual relationships (Schacht et al. 2017, 2022; Székely et al. 2014), intensifying male competition (Zhang et al. 2024) and sexual harassment (Hailey and Willemsen 2000; Le Galliard et al. 2005), we hypothesized ASR might similarly trigger germline‐mediated TGP. This was supported by correlative evidence (Hosseini et al. 2022) linking ASR biases to genome‐wide DNA methylation shifts in reproductive genes. However, offspring metamorphic traits (developmental speed, emerging weight, and stage duration) did not differ across ASR treatments in our standardized common garden. This indicates parental ASR exposure does not influence development via germline TGP, aligning with multigenerational null effects observed in *Callosobruchus maculatus* (Amiri and Bandani 2021). Thus, the applied ASR gradient or treatment duration may have been insufficient to induce stable, transgenerational epigenetic changes in the parental germline.

We also found no significant difference in brood ball weight across treatments, indicating that ASR did not drive shifts in gross parental investment via brood provisioning—the second core TGP pathway. Previous *O. taurus* studies demonstrate varied male effects on parental care; for instance, females paired with males (particularly horned males) often produce larger brood masses than solitary females (Hunt and Simmons 1998). Conversely, the heightened male numbers typical of male‐biased conditions could actively disrupt females during brood construction. The absence of weight differences in our study suggests males did not provide varying assistance across ASRs or that females exhibited compensatory behavior. In biparental species, female compensatory responses can significantly obscure variations in male care (Hunt and Simmons 2002b). Although unassisted *O. taurus* females increase their care effort, this compensation is often incomplete (Hunt and Simmons 2002b). In our experiment, females likely buffered or compensated for any ASR‐driven differences in male care, mitigating observable effects on final brood ball weight, consistent with recent ASR studies (Zhang et al. 2024). In summary, we found no evidence that ASR influences offspring development via either pre‐zygotic germline transmission or post‐zygotic gross nutritional provisioning, presumably because female compensation likely neutralized potential variation in male investment.

Although our results show no ASR‐induced TGP via germline modifications or gross nutritional provisioning, more cryptic transmission pathways may exist. In *O. taurus* , mothers can transmit environmental cues independently of gross provisioning by altering the physical architecture of the brood ball or secreting specific chemicals and hormones (Buzatto et al. 2012). Furthermore, females vertically transmit a highly specific microbiome exclusively through the maternal pedestal (Jones et al. 2025). While our artificial brood ball system successfully isolated the germline pathway, it inherently excluded the maternal pedestal and its associated secretions, severing these cryptic non‐nutritional transmission routes. The stark contrast between our null ASR results and the well‐documented density‐induced maternal effects (Buzatto et al. 2012) strongly suggests that different social stressors may utilize distinct TGP pathways. To disentangle these complex mechanisms, we recommend two specific, actionable directions for future research. First, conduct reciprocal egg transplants using natural brood balls across different ASR treatments to determine if ASR alters maternal investment via these cryptic pedestal modifications. Second, utilize our artificial system to selectively reintroduce specific microbial or chemical inoculations, thereby isolating precisely which maternal components mediate social‐environmental signals.

### 4.3 Recommendations for Future Research

To strictly isolate ASR‐induced TGP from genetic and demographic confounders, future studies must prioritize standardized experimental designs. First, because *O. taurus* is highly polyandrous (McCullough et al. 2017; Simmons and Holley 2011), ad libitum mating introduces paternity skew. Future work should implement restricted mating designs, confining females to mate with males of a consistent genetic background (Sato et al. 2023), to unambiguously attribute phenotypic differences to TGP rather than genetic variation. Second, to characterize parental sensitivity thresholds, researchers should employ finer gradients of both ASR and OSR to disentangle their independent and interactive effects. Third, multigenerational experimental designs are required to elucidate the persistence and cumulative evolutionary impacts of ASR‐induced transgenerational effects beyond the F1 generation. Fourth, because TGP may manifest behaviorally rather than morphologically (Amiri and Bandani 2021), future studies should incorporate offspring behavioral assays, such as testing aggression or mating competitiveness. Finally, closed experimental systems lack the ecological realism of ephemeral resources, inter‐patch dispersal, and interspecific competition (Moczek 2003). Utilizing semi‐natural mesocosms with mixed‐age populations or open‐field tracking will definitively test whether the developmental canalization observed here remains robust within a dynamic natural community.

To uncover the mechanistic underpinnings of offspring development, future work must incorporate targeted molecular analyses. Because ASR effects likely operate via gametic alterations, research should specifically investigate how social environments alter gamete quality and epigenetic composition. We recommend prioritizing the assessment of sperm‐mediated epigenetic changes driven by male–male competition, alongside maternal cytoplasmic inheritance vectors (Buzatto et al. 2012). Furthermore, future empirical and theoretical frameworks must integrate the relative contributions and potential interactive effects—whether synergistic or antagonistic—of “good genes”, TGP pathways, and within‐generation behavioral modifications on offspring fitness (Bonduriansky 2021; McAndry et al. 2025). To facilitate these ecological and mechanistic inquiries, we developed a novel egg–brood ball transplantation system. By encapsulating eggs, this method prevents the external environmental fluctuations and conspecific interference characteristic of previous open systems (Emlen and Nijhout 2001; Moczek and Nijhout 2002). Burying these structurally robust artificial brood balls in soil simulates natural subterranean conditions, providing a scalable, cost‐effective platform for large‐scale reciprocal transplants. However, while this extreme standardization successfully isolated germline‐mediated TGP from nutritional variations, it inherently stripped away complex, non‐nutritional components of parental care, such as maternal chemical secretions and microbiome inoculation. Omitting these factors may have biased our results toward a null finding. Therefore, we strongly recommend leveraging this artificial system in future studies to systematically reintroduce these specific variables. For example, comparing offspring reared in sterile artificial brood balls against those inoculated with specific microbial communities or natural maternal secretions will definitively untangle their relative contributions to offspring development. We suggest that this targeted methodological approach will be an invaluable tool for future research into dung beetle developmental ecology.

## 5 Conclusion

In conclusion, our results establish that *Onthophagus taurus* exhibits marked behavioral plasticity in response to ASR variation. Male‐biased environments maximized contest competition, while female‐biased conditions triggered peak courtship intensity, reflecting a strategic shift in male reproductive investment based on mate availability. Crucially, despite these profound parental behavioral adjustments, we found no evidence of ASR‐induced TGP via either core pathway. Our egg‐transplant protocol demonstrated that offspring development is robust to parental social history under standardized nutritional conditions, and consistent brood ball mass across treatments confirmed no ASR effects on post‐zygotic parental investment. Collectively, these findings reveal a decoupling between adult behavioral responses and intergenerational outcomes over short timescales: while adults rapidly adjust social interactions to prevailing ASR, offspring development appears canalized to ensure survival stability independent of parental social environment. Notably, our null result for ASR‐induced TGP contrasts sharply with well‐documented density‐induced maternal effects in this species, suggesting that TGP in dung beetles could be highly stressor‐specific, with distinct social stressors utilizing separate transmission mechanisms. Future work should combine standardized experimental designs with gametic epigenetic assays to determine whether social experience leaves molecular traces that manifest under alternative conditions or evolutionary timescales.

## Author Contributions

**Lisheng Zhang:** conceptualization (lead), data curation (lead), formal analysis (lead), methodology (lead), project administration (equal), resources (equal), software (lead), validation (equal), visualization (lead), writing – original draft (lead), writing – review and editing (lead). **Sudeshna Chakraborty:** data curation (supporting), formal analysis (supporting). **Tamás Székely:** conceptualization (supporting), funding acquisition (equal), investigation (equal), project administration (equal), resources (equal), supervision (equal), validation (equal), writing – original draft (supporting), writing – review and editing (supporting). **Jan Komdeur:** conceptualization (supporting), funding acquisition (equal), investigation (equal), project administration (equal), resources (equal), supervision (equal), validation (equal), visualization (supporting), writing – original draft (supporting), writing – review and editing (supporting).

## Funding

L.Z. was supported by a Ph.D. scholarship from the China Scholarship Council (CSC) and by the Dutch Science Council grant (ALW NWO ALWOP.531 and NWO VICI 823.01.014), and NWO TOP grant (854.11.003) to J.K. T.S. was funded by Nemzeti Kutatási, Fejlesztési és Innovációs Hivatal (NKFIH ADVANCED 150852, HU‐RIZONT‐2024‐00109 and HUN‐R) HUN‐REN‐Debrecen University reproductive strategies research group (Ref 110220).

## Conflicts of Interest

The authors declare no conflicts of interest.

## Supporting information


**Figure S1:** Offspring growth trajectories across adult sex ratio (ASR) treatments, split by offspring sex. Changes in body weight (measured every 48 h throughout development) are shown for female and male offspring separately across the three ASR treatments (FB: female‐biased; UB: unbiased; MB: male‐biased). Curves represent smoothed growth trends with 95% confidence intervals (shaded areas). Individual data points represent raw body mass measurements. Sample sizes (*n*) are indicated within each panel.
**Figure S2:** Offspring developmental duration across adult sex ratio (ASR) treatments, split by offspring sex. Duration of larval, pupal, and total emergence periods compared among ASR treatments (FB: female‐biased; UB: unbiased; MB: male‐biased) for female and male offspring separately. Box plots indicate the median (central thick line), interquartile range (box boundaries), and data range within 1.5 × IQR (whiskers). Individual data points are shown as dots. Sample sizes (n) are indicated within each panel.

## Data Availability

The data that support the findings of this study are openly available in Dryad at https://doi.org/10.5061/dryad.1g1jwsvbk.
