# Supplementary material for: Parental Social Environment Has no Effect on Offspring Development in the Dung Beetle: A Test of Adult Sex Ratio Effects
Source: Ecol Evol. 2026 Jul 6;16(7):e73833. doi: 10.1002/ece3.73833 (PMC13337320; doi:10.1002/ece3.73833)

**Appendixes**

**Figure S1**. Offspring growth trajectories across adult sex ratio (ASR) treatments, split by offspring sex. Changes in body weight (measured every 48 h throughout development) are shown for female and male offspring separately across the three ASR treatments (FB: female-biased; UB: unbiased; MB: male-biased). Curves represent smoothed growth trends with 95% confidence intervals (shaded areas). Individual data points represent raw body mass measurements. Sample sizes (n) are indicated within each panel.

**Figure S2**. Offspring developmental duration across adult sex ratio (ASR) treatments, split by offspring sex. Duration of larval, pupal, and total emergence periods compared among ASR treatments (FB: female-biased; UB: unbiased; MB: male-biased) for female and male offspring separately. Box plots indicate the median (central thick line), interquartile range (box boundaries), and data range within 1.5 × IQR (whiskers). Individual data points are shown as dots. Sample sizes (n) are indicated within each panel.

**Appendixes**

**Figure S1**.


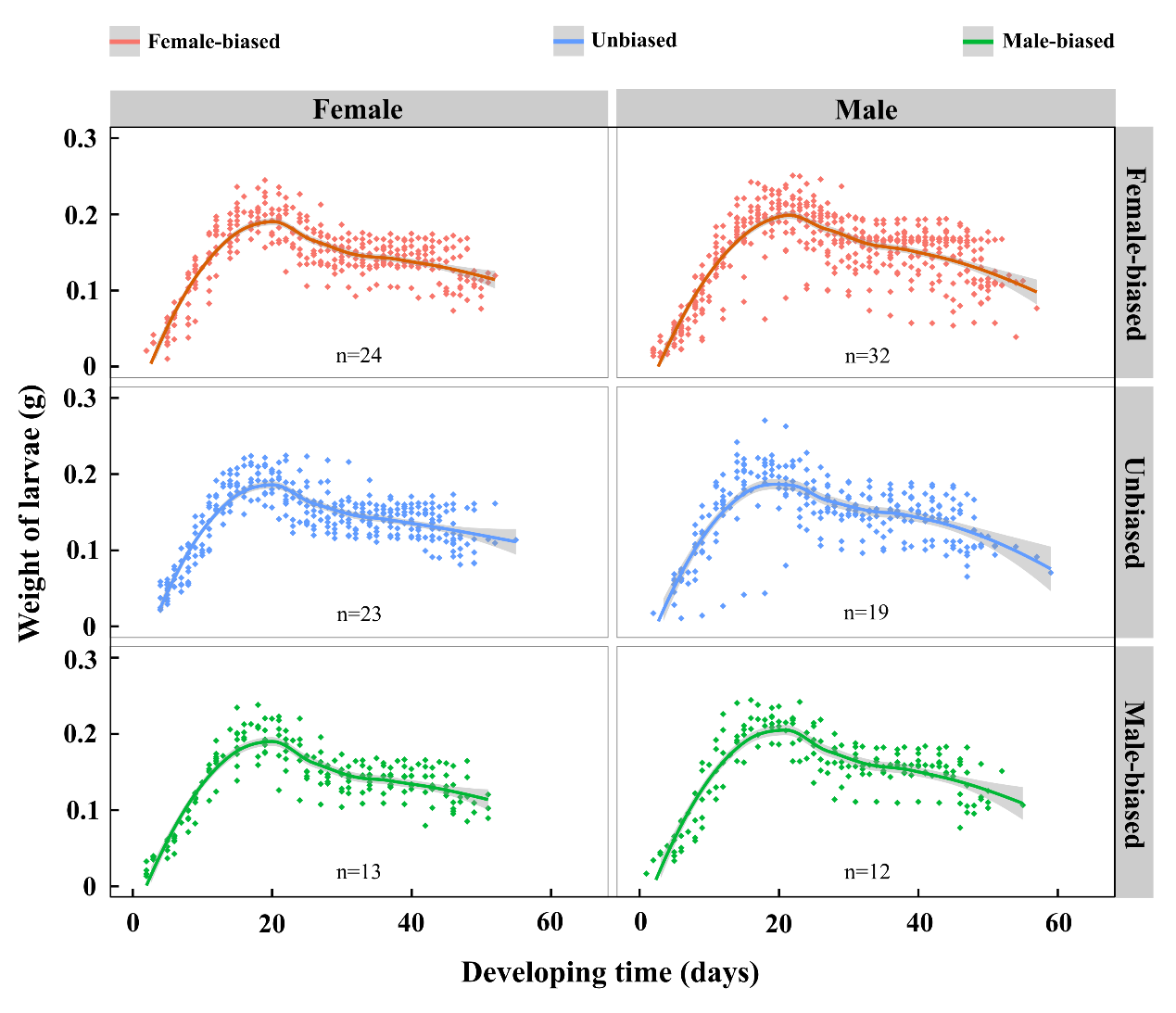


**Figure S2**.


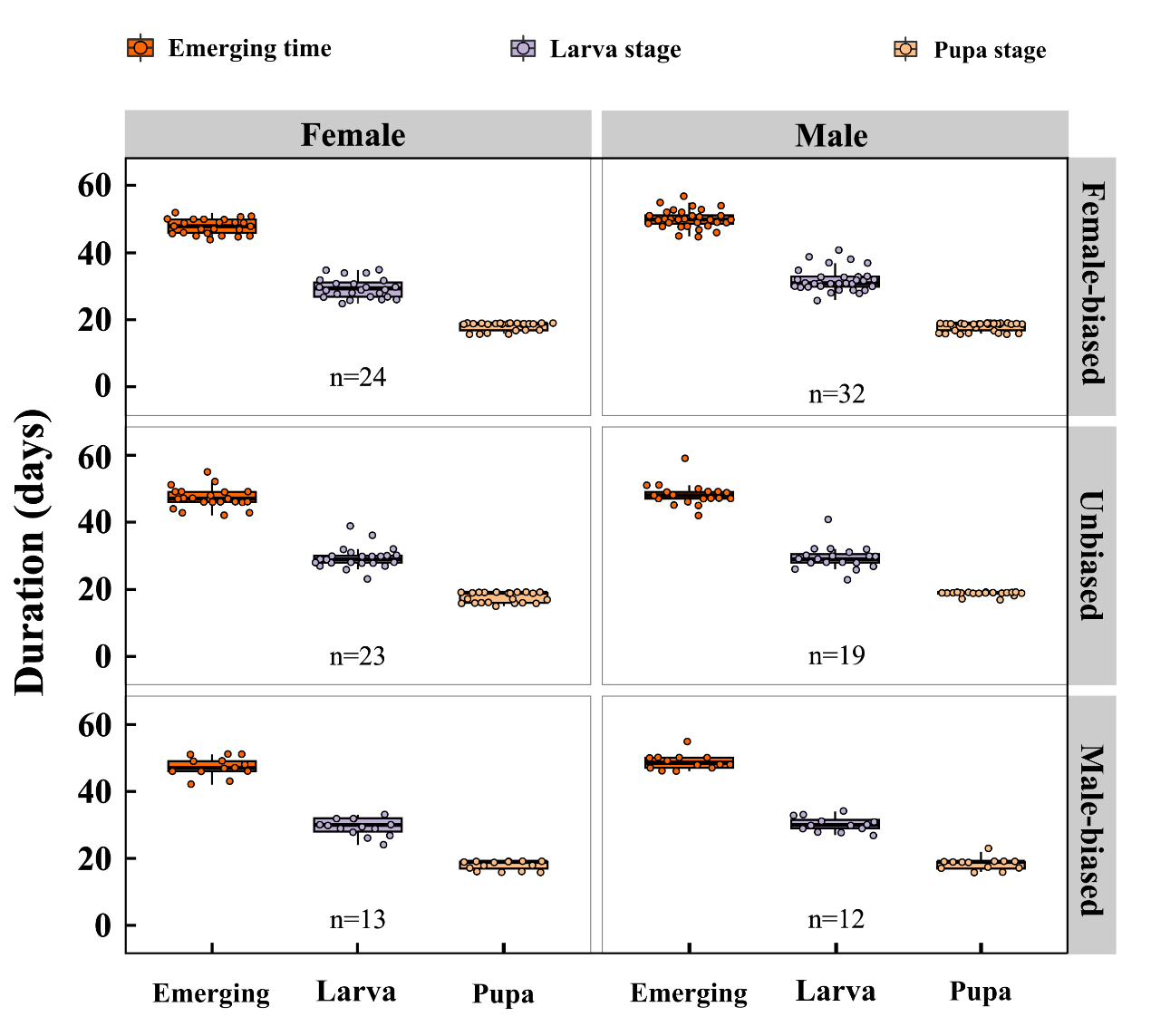

Supplement: Supplementary file 1 — Figure S1: Offspring growth trajectories across adult sex ratio (ASR) treatments, split by offspring sex. Changes in body weight (measured every 48 h throughout development) are shown for female and male offspring separately across the three ASR treatments (FB: female‐biased; UB: unbiased; MB: male‐biased). Curves represent smoothed growth trends with 95% confidence intervals (shaded areas). Individual data points represent raw body mass measurements. Sample sizes (n) are indicated within each panel. Figure S2: Offspring developmental duration across adult sex ratio (ASR) treatments, split by offspring sex. Duration of larval, pupal, and total emergence periods compared among ASR treatments (FB: female‐biased; UB: unbiased; MB: male‐biased) for female and male offspring separately. Box plots indicate the median (central thick line), interquartile range (box boundaries), and data range within 1.5 × IQR (whiskers). Individual data points are shown as dots. Sample sizes (n) are indicated within each panel. [file ECE3-16-e73833-s001.docx]
